# Evaluation of a meal replacement-based weight management program in primary care settings according to the actual European Clinical Practice Guidelines for the Management of Obesity in Adults

**DOI:** 10.1007/s00508-014-0585-6

**Published:** 2014-09-06

**Authors:** Renate Kruschitz, Sandra Johanna Wallner-Liebmann, Harald Lothaller, Maria Luger, Karin Schindler, Friedrich Hoppichler, Bernhard Ludvik

**Affiliations:** 1 Department of Endocrinology and Metabolism, Clinic for Internal Medicine III, Medical University Vienna, Währinger Gürtel 18–20, 1090 Vienna, Austria; 2 Institute of Pathophysiology and Immunology, Medical University Graz, Heinrichstraße 31a, 8010 Graz, Austria; 3 SIPCAN Special Institute for Preventive Cardiology and Nutrition, Guggenbichlerstraße 8/15, 5026 Salzburg, Austria; 4 Fischergasse14/II/12, 8010 Graz, Austria

**Keywords:** Weight loss program, Long-term effect, Primary care, Body composition, Obesity, Gewichtsreduktionsprogram, Langzeiterfolg, Niedergelassener Bereich, Körperzusammensetzung, Adipositas

## Abstract

**Introduction:**

The purpose of this study was the evaluation of a weight loss program in primary care settings with respect to the European Clinical Practice Guidelines for the Management of Obesity in Adults with regard to the long-term success of changes in body weight and composition.

**Methods:**

Overweight and obese patients (*n* = 1167) who underwent a standardized meal replacement-based weight loss program (myLINE^®^, AENGUS, Austria) in primary care settings were included in this evaluation. Body composition was measured by conventional anthropometry and bioelectrical impedance analysis (AKERN BIA101^®^, BIACORPUS RX4000^®^, SoftwareBodycomp Version 8.4 Professional). Data of patients who participated at least 12 months in the program were analyzed retrospectively and compared with their baseline data.

**Results:**

After 12 months, a weight loss of 8.6 ± 7.5 kg (mean ± standard deviation) or 8.2 ± 7.8 % from baseline was seen (*p* < 0.001). In all, 71.9 % of all patients achieved a minimal weight loss of 5 %, and 18.8 % lost 15 % of their initial weight. In comparison with the baseline (35.7 ± 11.5 kg), body fat decreased to 29.6 ± 10.7 kg, which is 83.7 ± 18.9 % from baseline (100 %; *p* < 0.001). Body cell mass showed an absolute reduction of − 1.4 ± 2.2 kg (*p* < 0.001), although a relative increase of 1.5 ± 2.5 % (*p* < 0.001). There were no significant differences between male and female subjects regarding changes in weight, body fat, and body cell mass.

**Conclusion:**

The evaluated program complies with the European Clinical Practice Guidelines for Management of Obesity in Adults (2008), which recommend a weight reduction of 5–15 % from initial weight within 6 months. Furthermore, the data showed a significant reduction of body fat and a relative increase of body cell mass.

## Abstract (de)

**Ziel:**

Evaluierung eines Adipositastherapieprogramms bezüglich der Konformität mit den European Clinical Practice Guidelines (2008) für das Management von Adipositas bei Erwachsenen und Kontrolle des Langzeiterfolgs anhand des Verlaufs von Körpergewicht und -zusammensetzung.

**Methodik:**

Übergewichtige Teilnehmer (*n* = 1167) eines standardisierten, ersatzmahlzeitenbasierten Adipositastherapieprogramms (myLINE^®^, AENGUS, Austria) wurden in österreichischen Arztpraxen ins Programm eingeschlossen. Die Körperzusammensetzung wurde mittels konventioneller Anthropometrie und Bioelektrischer Impedanz Analyse (AKERN BIA101^®^, BIACORPUS RX4000^®^, Software Bodycomp Version 8.4) erhoben. Die Daten jener Patienten, die zumindest 12 Monate im Programm waren, wurden retrospektiv analysiert und mit deren Ausgangsdaten verglichen.

**Ergebnisse:**

Nach 12 Monaten war ein Gewichtsverlust von 8,6 ± 7,5 kg (mean ± SD) bzw. 8,2 ± 7,8 % (*p* < 0,001) zu sehen. 71,9 % aller Teilnehmer erzielten eine Gewichtsreduktion von mindestens 5 und 18,8 % eine Gewichtsreduktion von 15 % ihres Ausgangsgewichts. Im Vergleich zum Körperfettanteil zu Programmstart (35,7 ± 11,5 kg) betrug der Körperfettanteil nach 12 Monaten 29,6 ± 10,7 kg, das sind 83,7 ± 18,9 % vom Ausgangswert ( = 100 %; *p* < 0,001). Während der 12 Monate kam es zu einer Abnahme der absoluten Körperzellmasse von − 1,4 ± 2,2 kg (*p* < 0,001), gleichwohl nahm die relative Körperzellmasse um 1,5 ± 2,5 % (*p* < 0,001) im Vergleich zum Programmstart zu. Es zeigten sich keine signifikanten Unterschiede zwischen den Geschlechtern hinsichtlich der Gewichts-, Körperfett- und Körperzellmasseentwicklung über den Beobachtungszeitraum.

**Schlussfolgerung:**

Das evaluierte Programm entspricht den European Clinical Practice Guidelines (2008) für die Therapie von Adipositas bei Erwachsenen, die eine Reduktion des Ausgangsgewichts von 5–15 % innerhalb von 6 Monaten empfehlen. Weiters zeigten sich eine signifikante Verringerung des Körperfettanteils und ein signifikanter Anstieg der relativen Körperzellmasse.

## Introduction

The current Austrian Nutrition Report shows an approximately 40 % prevalence of overweight and obesity among adults. Overweight is one of the most common health issues in Austria, as in many other European countries [1]. High body weight and fat are associated with an increased risk of cardiovascular complications, certain cancers, diabetes mellitus, Alzheimer’s disease, gallbladder disease, sleep apnea, osteoarthritis, renal disease, and musculoskeletal disorders [2–4]. A modest weight loss of 5–10 % has been shown to reduce the risk of developing diabetes by 58 % in at-risk patients [5] and reduces overall risk of mortality by 20 % [6–8]. Effective weight reduction can decrease disease risk, lower the health service expenditure, and improve the quality of life in affected persons [4, 9, 10]. Studies have shown that structured treatment programs with regular follow-up improve long-term weight loss and maintenance [11, 12]. Obesity management programs at primary care level should underlie regular quality checks [13, 14]. However, there is a lack of evidence about current approaches to obesity management in Austrian primary care.

In view of this fact, the present study evaluated the 12-month data based on the evaluation of changes in bodyweight and composition during the intervention and the follow-up phase of a standardized weight loss program conducted in primary care settings, according to the actual European Clinical Practice Guidelines for the Management of Obesity in Adults [15].

## Material and methods

### Subjects

A random sample of 1167 overweight participants (female = 979, male = 188; body mass index (BMI) ≥ 25 kg/m^2^) has been included in the weight loss program in 250 Austrian primary care settings.

Data of men and women who attended the program at least 12 months without interruption have been analyzed, and their results were compared with their baseline data. Exclusion criteria were BMI < 25 kg/m^2^, diabetes mellitus, being pregnant or lactating, cardiovascular disease, other medical conditions prohibiting weight loss, substance abuse, severe psychiatric illness, and eating disorders (anorexia nervosa, bulimia nervosa, binge eating disorder). The corresponding descriptive statistic is presented in Table 1.

**Table 1:** Descriptive characteristic at baseline

	Men	Women	Significance of differences^a^	Total
Number	188	979		1167
Age (years)	49.2 ± 13.8	46.5 ± 13.3	n.s.^b^	46.9 ± 13.4
Weight (kg)	107.5 ± 16.3	89.5 ± 17.7	*p* < 0.001	92.3 ± 18.7
BMI (kg/m^2^)	34.1 ± 5.0	33.0 ± 6.3	*p* = 0.004	33.1 ± 6.1
Fat mass (kg)	32.4 ± 9.9	35.9 ± 11.7	*p* < 0.001	35.7 ± 11.5
BCM (kg)	39.1 ± 5.9	26.3 ± 3.9	*p* < 0.001	28.5 ± 6.5

Data are mean ± standard deviation

*BMI* body mass index, *BCM* body cell mass

^a^Differences in the distributions of variables between men and women were tested by Student’s *t*-test for two independent samples (in case of normally distributed variables) and by Mann–Whitney *U*-test for two independent samples (if variables were not normally distributed)

^b^Not significant (*p* > 0.05)

The procedures used in this evaluation were in accordance with the Declaration of Helsinki (1964) and its later amendments. The study is approved and registered under No 2107/2013 of the ethics committee of the Medical University of Vienna. All participants gave their written informed consent prior to their inclusion into the program.

### Program design

The myLINE^®^-program (www.myline.at, AENGUS, Austria) is a standardized, meal replacement-based weight loss program for at least 24 weeks. The diet is in accordance with the latest nutritional recommendations of the German Nutrition Society (DGE), the Austrian Nutrition Society (ÖGE), the Swiss Nutrition Society (SGE), and the Swiss Association for Nutrition (SVE), which are called DACH-recommendations.

The program consists of four phases:

####  Starting phase:

During the first 2 days, the participants use exclusively a very low-calorie diet. Five meal replacements should be eaten on a regular basis every 3 h.

Total energy intake: 835 kcal/day, 60.0 g/day of protein, 15.5 g/day of fat, and 100.0 g/day of carbohydrates.

####  Reduction phase:

Beginning with the third day, two out of three meals should be replaced by an energy-reduced standard diet for weight control, and one meal should be eaten as regular fat-reduced food.

Participants are trained in creating their meal according to an exchange schema. The total fat content is ≤ 30 % of the daily energy intake. The reduction phase should be undertaken until the participant achieves two-thirds of the intended weight reduction, but at least for 10 weeks.

Total energy intake: 1000–1300 kcal/day, 50–65 g/day of protein (~ 20 energy percent (E%)), 34–44 g/day of fat (~ 30 E%), and 125–160 g/d of carbohydrates (~ 50 E%). For persons with a high body cell mass (BCM), the content of proteins would be individually adjusted.

####  Transitional phase:

The beginning of the transitional phase is determined individually, but the participant should have performed the phase 3 for at least 10 weeks. In this phase, at least one meal should be replaced and the other two should be eaten as a fat-reduced mixed meal.

Total energy intake: 1300–1600 kcal/day, 50–65 g/day of protein (~ 20 E%), 34–44 g/day of fat (~ 30 E%), and 160–200 g/day of carbohydrates (~ 50 E%). For persons with a high BCM, the content of proteins would be individually adjusted.

####  Stabilization phase:

Due to the acquired knowledge on how to arrange their meals correctly and how to adjust their energy intake to their daily requirements, the participants should maintain their weight without meal replacements. In this phase, they should eat three meals per day regularly.

During the whole program, at regular intervals (start, reduction, and transition phase: every 14 days; stabilization phase: once a month; afterward four times a year), the participants were measured by bioelectrical impedance analysis (BIA) and conventional anthropometry and received individual nutritional advice from a registered dietician. In the context of individual face-to-face consultations, aims and treatment plans, also including improvement of the activity level in accordance with the actual recommendations, are defined. A nutrition and activity diary for self-reflection and a participant handbook for support and exercises at home are used as instruments of behavior therapy.

After the weight reduction, there is the possibility to participate in a follow-up program. Regular controls should improve long-term weight loss and weight maintenance.

### Meal replacement

The declaration of all products took place at the Austrian Federal Ministry of Health as foods intended for use in energy-restricted diets for weight reduction. All products are in accordance with the directive 96/8/EG of the European dietetic food regulation. Furthermore, products undergo regular laboratory controls and are only available in primary care units in combination with regular follow-up examinations [16].

### Measure of body composition

The measurement of body composition was performed by conventional anthropometry (weight, height, waist circumference) and BIA (AKERN BIA 101^®^, BIACORPUS RX 4000^®^, Software Bodycomp version 8.4 professional).

### Statistics

Statistical analysis was performed by SPSS for Windows Version 17.0 (SPSS Inc., Chicago). All results are presented as mean ± standard deviation (SD). Differences in the distributions of variables between men and women were tested by Student’s *t*-test for two independent samples (in case of normally distributed variables) and by Mann–Whitney *U*-test for two independent samples (if variables were not normally distributed). Comparison between baseline and 12 months was done using analysis of variance. *p*-Values < 0.05 were considered as significant.

## Results

All participants who underwent the program for at least 12 months without interruption were included in the evaluation. Their baseline characteristics are presented in Table 1. Men showed a higher weight (*p* < 0.001), BMI (*p* < 0.001) and muscle mass (*p* < 0.001) and lower fat mass (*p* < 0.001) in comparison with women at baseline, although there were no significant differences between male and female in the development of weight, body fat, and BCM during the observation period.

After 12 months, a weight loss of 8.6 ± 7.5 kg (mean ± SD) or 8.2 ± 7.8 % was seen (*p* < 0.001; Table 2), which corresponds to a loss of excess weight of 28 %. In all, 71.9 % of all participants reached a minimal weight loss of 5 %, 40 % lost 10 %, and 18.8 % of the participants lost 15 % of their initial weight. These findings correspond to a mean BMI reduction of 3.1 ± 2.7 units (*p* < 0.001; Table 2). A total of 81.5 % of the participants achieved a minimum BMI reduction of 1 unit after 12 months. Body fat decreased by 6.2 ± 5.9 kg (*p* < 0.001; Table 2). In comparison with baseline data of 35.7 ± 11.5 kg, body fat decreased to 29.6 ± 10.7 kg after 12 months, which is 83.7 ± 18.9 % from baseline (100 %; *p* < 0.001; Table 2). With regard to the time course of weight loss, we could obtain data from a smaller sample (70 from 1167 patients), which showed that the maximum body weight and body fat reduction was achieved after 6 months. Furthermore, BCM showed an absolute decrease of − 1.4 ± 2.2 kg (*p* < 0.001), but a relative increase of 1.5 ± 2.5 % (*p* < 0.001) after 12 months (Table 2).

**Table 2:** Body composition data from baseline to 12 months

*N* = 1167	Baseline	12 months	Differences between BL and 12 months	Significance of differences^a^
Weight (kg)	92.3 ± 18.7	83.7 ± 16.8	− 8.6 ± 7.5	*p* < 0.001
BMI (kg/m²)	33.1 ± 6.1	30.0 ± 5.5	− 3.1 ± 2.7	*p* < 0.001
Fat Mass (kg)	35.7 ± 11.5	29.6 ± 10.7	− 6.2 ± 5.9	*p* < 0.001
Fat Mass (%)	38.2 ± 6.9	34.7 ± 7.3	− 3.5 ± 3.9	*p* < 0.001
BCM (kg)	28.5 ± 6.5	27.1 ± 6.1	− 1.4 ± 2.2	*p* < 0.001
BCM (%)	31.2 ± 4.8	32.7 ± 5.0	1.5 ± 2.5	*p* < 0.001

Data are mean ± standard deviation

*BL* baseline, *BMI* body mass index, *BCM* body cell mass

^a^Comparison between baseline and 12 months was calculated using analysis of variance

## Discussion

Successful weight reduction and weight maintenance after weight reduction is the greatest challenge in the therapy of obesity. Effective methods to achieve this ambitious goal, especially in primary care, are required [17, 18]. In this study, a meal replacement-based weight management program was evaluated according to the actual European Clinical Practice Guidelines for the Management of Obesity in Adults, with respect to the long-term success regarding changes in body weight and body composition. As defined by the European Guidelines, a weight reduction of 5–15 % within 6 months or 0.5–1.0 kg per week is realistic and desirable [15]. International committees consider a weight reduction of 5–10 % of the initial body weight as adequate to reduce the health risk in obese patients [15, 19]. Additionally, Hauner et al. [13] defined success for obesity therapy programs in primary care as follows: at least 50 % of the participants have to lose 5 % of their initial weight, and 20 % should achieve a weight reduction of 10 % from baseline. For the definition of long-term success, there exists no uniform definition. The Institute of Medicine defines long-term success as an achieved body weight at least 5 % below the initial weight or a BMI at least one unit below the baseline BMI 1 year after weight reduction [20]. Wing and Hill [21] proposed that successful weight loss maintainers are defined as patients who initially have lost at least 10 % of their body weight and stabilized at least for 1 year. The Clinical Guidelines on Evaluation and Treatment of Overweight and Obesity in Adults have defined successful weight maintenance after weight loss as a weight regain of less than 3 kg in 2 years [22].

In the case of the evaluated program, the average weight reduction was 8.6 kg or 8.2 % after 12 months. Compared with the data of Franz et al. [23], which systematically reviewed the long-term effect of 80 different medical weight loss interventions, the present data are well within or even above the reported range. In the current study, approximately three-quarter of the participants reached a weight reduction of 5 %, and 40 %, a reduction of 10 %. Due to the program structure, it is subsequently not possible to provide an accurate retention rate, as participants may achieve their weight goal before 12 months or some may stop the program after a shorter period than 12 months and restart it again afterward, which can therefore not be defined as dropouts.

BMI after 12 months was, on average, 3 units lower compared with the BMI at the beginning of the program. These results are in accordance with the criteria referred earlier in the text. Long-term weight loss was greater than weight loss observed in other primary care trials [24–27], with the exception of a study involving morbidly obese patients who were treated with intensive group lifestyle modification and weight loss medications [28].

Reduction in body fat and preservation of lean body mass are crucial for beneficial metabolic effects and prevention of weight regain and sarcopenic obesity due to loss of BCM [18]. White adipose tissue is recognized as an active endocrine tissue that secretes numerous immunomodulatory factors that play major roles in regulation of human metabolic and vascular biology, and is therefore significantly involved in the pathogenesis of the metabolic syndrome [29]. The collected data showed a body fat reduction of 16 % from baseline to 12 months, which suggests a metabolically effective weight reduction, which we cannot prove due to the lack of respective labor parameters. During the observation period, we could observe an increase of relative BCM despite a decrease of absolute BCM indicating a satisfactory overall development of muscle mass despite weight reduction.

A possible explanation for the success of the examined weight management program may be the intensive patient contact, the individual dietary counseling, and the guidance for physical activity, as well as the standardized optimal nutrient intake. One essential component of the program is a long-term modification of dietary and physical activity habits. The program supports the participants during and after the weight reduction with behavior-therapeutical modules as a nutritional and physical activity diary, for patient self-monitoring and as  a basis for the therapeutic one-on-one counseling, through a participant handbook and group meetings, as a communication platform for participants, and for increasing their nutritional knowledge. In further studies, the quality of participant contact and participant satisfaction in this program was evaluated [16, 30–32], and therefore, the program complies with the requirements of regular quality checks of obesity programs in primary care [13].

## Conclusion

The evaluated program complies with national and international guidelines for the therapy of obesity in adults and is efficient with regard to long-term therapeutic use in primary care. The results emphasize the importance of regular body composition measurements during weight reduction. The findings reveal a significant reduction of body weight, body fat, and a relative increase of BCM after 12 months. Participants underwent regular controls with conventional anthropometry, BIA measurements, and dietary advice from a registered dietician, and, in addition, behavior-therapeutical tools supported them during and after weight reduction. The program represents an intensified conventional obesity therapy with ongoing quality checks. Further studies with an observation period of more than 12 months in consideration of metabolic parameters are needed to examine the long-term weight development and the metabolic effect of this weight management program.

### Conflict  of interest

Sandra Johanna Wallner-Liebmann and Bernhard Ludvik provide gratuitous service for the companies’ scientific advisory board. Renate Kruschitz, Maria Luger, Karin Schindler, and Friedrich Hoppichler declare that they have no conflict of interest.
